# HPV Vaccination in Romania: Attitudes, Practice, and Knowledge Among Frontline Healthcare Providers

**DOI:** 10.3390/microorganisms14010205

**Published:** 2026-01-16

**Authors:** Maria Moise-Petu, Lacramioara Aurelia Brinduse, Eugenia Claudia Bratu, Florentina Ligia Furtunescu

**Affiliations:** 1 Discipline of Public Health and Management, Faculty of Medicine, Carol Davila University of Medicine and Pharmacy, 020021 Bucharest, Romania; lacramioara.brinduse@umfcd.ro (L.A.B.); eugenia.bratu@umfcd.ro (E.C.B.); florentina.furtunescu@umfcd.ro (F.L.F.); 2 National Institute of Public Health, 050463 Bucharest, Romania

**Keywords:** vaccination, human papilloma virus (HPV), vaccine coverage, cervical cancer, trust in vaccination, training needs

## Abstract

Recognizing cervical cancer as a major public health concern, Romania was among the first EU countries to introduce human papilloma virus (HPV) vaccination in 2008. Despite multiple strategies implemented over the past 17 years, HPV vaccine coverage remains one of the lowest in the EU, while cervical cancer mortality rates are among the highest. To explore the underlying factors, we conducted a cross-sectional study involving 209 family physicians at the national level. The study assessed their attitudes, practice, knowledge, and training needs related to HPV vaccination. The majority of physicians (90%) reported that they provide HPV vaccination services, and 88.5% considered themselves to have good and very good knowledge about HPV, which they routinely share during consultations with patients. However, respondents noted that both physician and public attitudes toward HPV vaccination are only moderately positive, which limits vaccine uptake and the success of prevention efforts. Parental hesitation was the main barrier, mentioned by 81.8% of respondents. The majority (71.3%) of doctors indicated that they were able to adequately respond to patients’ questions, but 81.4% of respondents expressed the view that additional training is needed for healthcare professionals on HPV infection and vaccination. These findings highlight the need for coordinated efforts to increase demand and trust in HPV vaccination. Recommended strategies include targeted professional training, public information campaigns, and the development of strong cross-sector partnerships to support vaccination efforts.

## 1. Introduction

Cervical cancer (CC) remains a significant public health challenge worldwide, despite notable progress in prevention, particularly in high-income countries. Globally, cervical cancer ranks as the eighth most common malignant neoplasm among women. In 2022, an estimated 660,000 new cases and approximately 350,000 deaths occurred worldwide [1,2]. Persistent infection with high-risk human papillomavirus (HPV) types is recognized as the main etiological agent of cervical cancer. Although more than 200 HPV types have been identified, only 14 are considered oncogenic, with HPV types 16 and 18 accounting for approximately 71% of cervical cancer cases, followed by types 31, 33, 45, 52, and 58, which contribute to a further 18% [3].

In the European Union (EU), cervical cancer is the second most frequent malignancy in women aged 15–44, with an estimated 33,000 new cases and 15,000 deaths reported annually [4]. In 2020, cervical cancer ranked as the third leading cause of cancer-related death in young European women (15–44 years) [5].

Romania continues to report some of the highest cervical cancer incidence and mortality rates in Europe. In 2022, the age-standardized incidence rate of HPV-associated cervical cancer in Romania was 21.7 per 100,000 women (with a total of 3368 new cases) versus 10.6 per 100,000 women, (with a total of 58,219 new cases) in Europe. The mortality rate was also high, with an age-standardized rate of 9.3 per 100,000 women (crude rate: 18.3), accounting for 1793 deaths, in Romania versus 3.9 per 100,000 women (crude rate: 7), accounting for 26,950 deaths, in Europe [2]. In 2023, 1177 deaths caused by malignant cervical tumor were recorded at the country level, with an overall mortality rate of 5.4/100,000 inhabitants, representing 0.5% of total deaths and 2.5% of deaths from tumors [6]. Cervical cancer is currently the third most common cancer among Romanian women and the second among women aged 15–44, making it the fourth overall and second most frequent cause of cancer-related mortality in this age group [7].

Persistent infection with oncogenic HPV types is the primary cause of over 95% of cervical cancer cases [1,8]. Approximately 90% of HPV infections in women are naturally cleared within 1–2 years; however, the likelihood of viral persistence tends to increase with age [9]. HPV is also implicated in other anogenital and oropharyngeal cancers, including penile, vulvar, anal, and a subset of head and neck cancers. Low-risk HPV types are responsible for benign lesions such as genital or oral warts, oral epithelial hyperplasia even in pediatric subjects, and recurrent juvenile respiratory papillomatosis [3,5,10,11]. Given the multifactorial burden associated with HPV, the implementation of effective primary prevention strategies, including vaccination, remains a critical component of public health efforts.

The availability of HPV vaccines highlights the need for comprehensive primary prevention strategies that transcend gender- or age-specific limitations.

Over the past 17 years, Romania has implemented various national strategies for HPV vaccination, but coverage remained low, with limited uptake over the years. The first publicly funded, school-based vaccination program was launched in 2008 through the National Oncology Program [12,13]. In 2017, a new approach was introduced, whereby HPV vaccination was offered free of charge upon parental request, targeting girls aged 11 to 14 years, primarily administered through family medicine practices [14]. In October 2021, the Ministry of Health expanded the eligibility criteria for free HPV vaccination to include all girls aged 11 to 18 years under the national program for risk groups [15]. A significant change in the accessibility of the HPV vaccine occurred, starting from December 2023, with its inclusion in Romania’s national reimbursement system. This transition marked a major legislative and logistical change, as the Ministry of Health (MoH) ceased the centralized procurement of HPV vaccine doses. Instead, the vaccine became available in community pharmacies, based on individual prescriptions. The cost of the vaccine is now covered by the National Health Insurance Fund. A regulatory protocol was introduced governing eligibility for vaccination, stipulating that full reimbursement is available for girls and boys aged 11 to 18, while women aged 19 to 45 are eligible for a 50% reimbursement [16,17]. In October 2025, the age categories changed as follows: full reimbursement for girls and boys aged ≥11 to <27, 50% reimbursement for women aged ≥27 to <46 years [18,19].

From 2020 to the present, the HPV vaccine that is recommended and available in Romania is only the 9-valent one, which covers HPV serotypes 6, 11, 16, 18, 31, 33, 45, 52, and 58 [20]. Regarding the last seven strains included in the vaccine, these are the same high-risk serotypes that are most commonly involved in cervical cancer in all regions of the world in different proportions [5,21]. The other two low-risk HPV types, 6 and 11, are commonly involved in benign lesions [4,5]. Data regarding the most frequent HPV oncogenic types in Romania among women are very limited. We do not have prevalence data for the serotypes involved in cervical cancer. The published data refer mainly to serotypes frequently involved in low-grade and high-grade lesions (16, 18, 31, 33, 51, 58, 45), which are the same, but occur in different proportions [7]. These strains are also involved in high-grade lesions in the European region and worldwide [5,21]. To benefit from the availability of the vaccine, which provides the best coverage and most extensive protection against HPV-related diseases [20], combined with increased accessibility, it is very important to assess the level of knowledge regarding HPV infection and HPV vaccination among both doctors and the general population.

Our study aims to assess the attitudes, practices, and knowledge of family physicians, the main vaccinators in Romania. Given their major role in vaccination, understanding the level of knowledge, attitude, and practice of family doctors, as well as the challenges they face, is essential for increasing vaccination in the future. We need to analyze what has changed positively over the years of practice regarding the use of HPV vaccine for cervical cancer prevention, while at the same time clearly documenting the shortcomings that hold us back in terms of vaccination coverage. Only in this way will we be able to contribute to the development of personalized information packages and communication programs in primary health care, which harmoniously complement the expanded access to vaccination, continuously supporting a well-developed strategy at both national and global levels to achieve the WHO target of 90% complete vaccination coverage of girls by 15 years of age, which is a key objective to eliminate cervical cancer as a public health problem.

## 2. Materials and Methods

### 2.1. Study Design

An observational, descriptive, cross-sectional study was conducted at the national level among family physicians in Romania. A relevant professional organization was involved in the dissemination of the study questionnaire using the snowball strategy. The national association sent the tool to its county branches, which distributed it to the family doctors in that geographical area. The only selection criterion for respondents was practicing as a family physician.

A total of 213 healthcare professionals completed the questionnaire: 4 of them were excluded from the analysis, 2 as they were doctors from a specialty other than family medicine and 2 as they were nurses.

### 2.2. Data Collection

Data were collected through a structured questionnaire composed of 37 mixed-format items, organized into three thematic sections. The first section covered socio-demographic characteristics (6 items), the second addressed logistical and administrative aspects related to vaccination and HPV vaccination (8 items), while the third focused on participants’ attitudes, perceptions and approach to practice, knowledge, and challenges concerning HPV vaccination (23 items). The questionnaire employed various item formats, including closed-ended questions with single or multiple pre-formulated response options, open-ended questions, dichotomous (yes/no) questions, and general items—particularly those investigating sources of information about HPV vaccination—featuring both structured and unstructured response formats. Some questions were measured on a 6-point Likert scale: “in favor of vaccination”, “rather in favor of vaccination”, “partially in favor of vaccination”, “rather disapprove of vaccination”, “disapprove of vaccination”, and “unspecified”. For the knowledge question linked with information regarding vaccination, a 5-point scale was used: very important = 4; important = 3; neutral = 2; less important = 1; not at all important = 0.

Data collection was conducted via Google Forms, a cloud-based survey platform, with the questionnaire remaining accessible online for a period of three months, from February to April 2024. Upon accessing the survey, participants were provided with detailed information regarding the objectives of the study and were required to provide informed consent before proceeding. The completion of all questionnaire items was mandatory, and submission was only possible once all sections were fully completed. Participation was entirely voluntary and anonymous.

We used, as a basis, a questionnaire developed and applied within the framework of the European project Partnership to Contrast HPV (PERCH) [22], implemented in 18 European countries, including Romania, dedicated to assessing training need. The instrument was adapted to include questions related to attitudes, perceptions, knowledge, and practices specific to Romania. The study was approved by the Scientific Research Ethics Committee of the “Carol Davila” University of Medicine and Pharmacy, under approval number 3591, issued on 12 February 2024.

### 2.3. Statistical Analysis

The data collected were compiled and organized using Microsoft Excel. Statistical analyses were subsequently performed using IBM SPSS (Statistical Package for Social Sciences) Statistics software for Windows (29.0 version; Armonk, NY, USA: IBM Corp).

Descriptive analyses were performed to compare the main characteristics of medical doctors from rural and urban areas. The analysis focused on attitude and perception of HPV vaccination, practice and knowledge, and the need for continuous medical education. Independent variables included the administrative–territorial affiliation of the medical office, professional rank, gender, and urban versus rural distribution.

For the quantitative variables, the distribution was analyzed using the Kolmogorov–Smirnov test for normally distributed variables and median for other scale variables. The comparisons between urban and rural area and between male and female were analyzed using parametric (*t*-test) or non-parametric (Mann–Whitney test) tests according to the distribution of variables. The qualitative variables were presented as counts and percentages, and the distribution between urban and rural areas was analyzed using the chi-square test. The threshold of statistical significance was *p* < 0.05.

## 3. Results

### 3.1. Demographic, Logistical, and Administrative Characteristics

A number of 209 family physicians were retained in our study, with 66.0% from urban and 34.0% from rural areas. The female gender dominated in the study group, being represented by 85.6% of the participants. The age of the respondents ranged from 35 to 73 years, with an average age of 56.0 years. Most of them, 68.9%, were consultant physicians and a majority, 90.0%, worked in an individual office, geographically distributed in 37 out of the 41 counties of Romania, in addition to the capital, Bucharest. Among those who indicated a number of registered children, we noticed huge variations, from a minimum of 5 children to maximum of 2000, with a median of 311.5 (Q1–Q3) children. No statistically significant difference was observed between urban and rural areas for all these general characteristics, except for professional rank (*p* < 0.001). The distribution of general characteristics among urban and rural areas is represented in Table 1.

### 3.2. Attitudes and Perceptions

Attitude towards and perception of vaccination were approached from two perspectives: personal perception, regarding one’s own behavior, and perception of others (health professionals and population). When considering the personal perspective, the majority of family doctors, 95.2%, mentioned that they are in favor of vaccination in general and 93.3% were in favor of HPV vaccination. Few doctors had a moderate attitude towards vaccination (4.3% “rather” in favor and 1 “partially” in favor). Similarly, for HPV vaccination, 3.8% and 1.9% of the doctors reported they are “rather”, and “partially” in favor of HPV vaccination, with two doctors disagreeing with it. When discussing the attitude of other doctors towards HPV vaccination, the respondents’ perception was more reserved. Only 25.8% of family physicians who participated in the study noted a favorable attitude of doctors regarding HPV vaccination, while 36.8% of them held a rather favorable and 33.0% a partly favorable attitude.

Regarding the population’s attitude towards HPV vaccination, only 1% of family physicians mentioned that the attitude of the population is in favor of HPV vaccination, with 10.5% of them perceiving a rather favorable attitude and 65.1% a partly favorable attitude among the population. Family doctors from urban areas consider the population’s attitude towards HPV vaccination to be better (*p* = 0.049), as shown in Table 2.

### 3.3. Practice Approaches

A total of 97.6% of doctors mentioned that they practice vaccination according to the National Immunization Calendar (NIC) for children (**N** = 204 responders). Also, 90.0% of responders declared that they usually vaccinate against HPV (**N** = 188). We recorded a statistically significant difference (*p* = 0.004) between administrative–territorial units, with 94.2% of family physicians in urban areas and 81.7% in rural areas vaccinating against HPV, as shown Table 3.

Regarding the number of doses of HPV vaccine administered in 2022, the last full year in which vaccine procurement was performed centrally by the Ministry of Health, numbers ranged from 1 to 200 among the 188 doctors who answered yes to this question. A statistically significant difference (*p* < 0.001) was registered between administrative-territorial units, with a median (Q1–Q3) of 20 doses for urban areas, and a respective 9.5 doses for rural areas. The group eligible for free HPV vaccinations in 2022 included girls between 11 and 18 years old, based on requests made by parents or caregivers.

There was also a statistically significant difference in recommendation for vaccination at the age of 18 and over between urban and rural areas (*p* = 0.046).

Almost all, 99.0%, of family doctors stated that they provide information to parents, children, and adolescents about HPV infection and HPV vaccination, especially to mothers (47.8%), followed by children/teenagers and parents together (35.4%), and both parents (13.9%). Nurses also contributed to the educational discussion, as mentioned by 45.9% of the respondents. Regarding the people most interested in discussing HPV and vaccination and who asked the most questions, mothers came first (85.6%), followed by young adults (26.3%), and both parents equally (7.7%).

Educational discussion should be a regular activity, as considered by 51.2% of respondents, with a similar percentage encouraging discussion when checking the vaccination schedules of new patients. This was a question that allowed for the selection of multiple answers.

The main sources of information about HPV and HPV vaccination were continuing medical education courses and events (81.3% of doctors), followed by information, notes, or guidelines published by medical associations and medical society websites (70.3%). Family doctors in urban areas access the websites of international organizations to a greater extent than those in rural areas, with a statistically significant difference (*p* = 0.025), Table 4.

The main difficulties encountered by family physicians in initiating and completing the HPV vaccination scheme were parental hesitation, mentioned by 81.8% of respondents, followed by the high price of vaccines in pharmacies, mentioned by 53.6% of physicians from urban areas and 40.8% from rural areas. Other difficulties were delays in vaccine supply (51.4% in urban areas and 40.8% in rural areas), difficulties in counseling parents and children (33.3% in urban areas and 45.1% in rural areas), a lack of vaccine doses in clinics (23.2% in urban areas and 15.5% in rural areas), a lack of doses in pharmacies (10.9% in urban areas and 4.2% in rural areas), and difficult registration mechanisms (4.3% in urban areas and 5.6% in rural areas). There were no statistically significant differences between urban and rural areas for any type of difficulty.

### 3.4. Knowledge

Most doctors considered themselves to have a good (60.3%) or very good (28.2%) level of knowledge about HPV; also, 71.3% of them were able to successfully answer patients’ questions. Only 11% of them noted a partial level of knowledge.

The main topics that challenged the doctors when responding accurately were related to efficacy in boys for 28.2% of them, contraindications for 26.8%, safety for 26.3%, and evolution of infection for 20.6%. We obtained a statistically significant difference between urban and rural areas (*p* < 0.001) for contraindications, respective to HPV distribution and transmission (*p* = 0.048). Other topics that family physicians could not answer accurately are mentioned in Table 5.

More than half of the respondents, 58.4%, mentioned that they had not attended any training courses on HPV infection/HPV vaccination in the last 5 years. We found that only 24.4% of family doctors were exposed to information about HPV and HPV vaccination within a specialty training program (residency, postgraduate studies). An important proportion of family physicians, 81.4%, mentioned an interest in participating in future training on HPV, and 12.4% were irresolute.

The respondents’ level of knowledge was also investigated through essential information that should be included in training material dedicated to HPV and vaccination. We performed a statistical analysis of each topic according to gender and area of residence, based on scoring. Female doctors from urban areas considered information regarding HPV distribution and transmission (*p* = 0.003), and the evolution of HPV infection and burden of disease (*p* = 0.005), to be more important than male doctors, with the observed differences being statistically significant. Female doctors from rural areas considered information regarding benefits and risks at 18 years and older (*p* = 0.033), advice and counseling for parents and their children (*p* = 0.027), advice and counseling for adolescents (*p* = 0.004), and counseling sessions with skeptical parents to be more important than male doctors (*p* = 0.049), as shown in Table 6.

## 4. Discussion

HPV vaccination coverage has remained an important public health issue for many years in many parts of the world, and particularly in Romania. In most countries around the world, universal HPV vaccination programs are fully publicly funded and are provided primarily in schools or through a combination of health clinics and school programs [23].

Although the vaccination program was introduced relatively early in Romania, in 2008, it was not successful, being initially implemented at the school level. There are countries where this approach has worked very well [24] and even the latest scientific evidence mentions that in areas with high HPV vaccination uptake, vaccinations took place mainly in schools, while in areas with low vaccination uptake, vaccination was administered mainly in health centers or private medical offices [25]. Another study suggests that HPV vaccination in schools is more cost-effective than vaccination outside of school, from both the payers’ and societal perspectives, when taking into account the better vaccination coverage, which is important in achieving herd immunity [26].

In our country, this first experience created a continuous chain of challenges through the distrust created among both the population and doctors, giving way to the spread of erroneous information, not based on scientific evidence. Subsequently, the vaccination program was carried out at the primary healthcare level, and was suspended and resumed several times due to low vaccination rates. Year after year, health professionals and authorities, as well as part of the population, have sought to understand the phenomenon that was created and have tried to find optimal solutions for both access and education regarding cervical cancer prevention.

Providing the HPV vaccination program through family physicians has been a justified approach. These health professionals play a significant role in setting standards and enjoy a high level of public trust, being able to provide advice to emphasize the importance of HPV vaccination [27,28]. In Romania, family physicians are the main vaccinators for vaccines included in the NIC, vaccines addressed to high-risk groups, and optional vaccines. France is facing the same situation, with the main vaccinators being general practitioners [28]. This raises the issue of the level of training and the need for regular training to increase their ability to provide recommendations for HPV vaccination [26].

Although the scientific evidence accumulated over the last 19 years of the practical use of HPV vaccination is vast, consistently highlighting the positive benefit–risk ratio, the same two common reasons for non-vaccination remain constant: insufficient knowledge and fear of the potential negative effects of the vaccine [26], likely supported by environmental influences spreading false information about vaccines [29].

Over time, various studies have attempted to document the knowledge of, attitude towards and perception of HPV infection and HPV immunization or the challenges related to this topic among physicians, medical students and parents/women in Romania, in the same research or in separate research on each population category [27,30,31,32,33,34]. None of these studies were conducted specifically among family physicians at the national level.

However, the attitude, knowledge, perception, and training needs of both parents and eligible individuals, as well as health professionals, must be carefully and periodically assessed to increase the positive aspects that are currently recorded in terms of vaccination coverage—which increased from 2,5% in 2008 [12] to 23% in 2023, with a slight downward trend in 2024 to 17% for the full vaccination schedule in women [35]—into a real success in cervical cancer prevention. It is important to get closer, year by year, to 90% vaccine coverage for girls by the age of 15, which is one of the WHO goals of eliminating cervical cancer as a public health problem by 2030, included in the National Vaccination Strategy 2023–2030 [36].

In the current framework of HPV vaccination, provided via reimbursement mechanism through electronic prescriptions issued by physicians under contract with the National Health Insurance House, it is essential to prioritize both key stakeholders involved in the immunization process: the recipient and the prescriber.

Family physicians are the main prescribers of the HPV vaccine and vital advocates for patients, but studies indicate that they frequently face vaccine-related myths [37] and hesitant patients [38]. This means that they must be very well equipped in terms of the scientific arguments related to HPV vaccination and their ability to convey confidence in this important preventive measure.

Of the 209 family doctors included in the study, the majority, 85.6%, were women, as most doctors in Romania belong to this gender, an aspect that is also reflected in family medicine according to statistics. In line with the National Institute of Statistics, in Romania there were 12.600 family doctors in 2024, of which 9.789 were women, representing almost 78% of the total in this specialty [39]. Of these, only 10,470 were in a contractual relationship with the National Health Insurance House (NHIH) in the first quarter of 2024 and could provide vaccination services [40].

In our study, the majority of family physicians reported that they are in favor of vaccination in general (95.2%) and of HPV vaccination in particular (93.3%), in line with our expectations. These percentages, however, are not reflected in the vaccine uptake rate. A similar situation was described in a study involving dental students: although 96.9% had information about the safety and effectiveness of HPV vaccination in preventing cancer, only 10.6% of them were vaccinated [41]. Surprisingly, the study published by Karafillakis in 2019 mentioned that in the European region, including Romania, there is the lowest level of trust in vaccination, especially regarding the safety of vaccines [42].

Family physicians’ perception regarding the attitude of Romanian doctors towards HPV vaccination was very different from their own reported attitude, with 25.8% mentioning a favorable attitude of the doctors, irrespective of specialization, 36.8% a rather favorable, and 33.0% a partly favorable attitude. These findings are slightly different from patients’ perceptions of doctors’ opinion regarding the need for HPV vaccination for the year 2023 at the national level, where 38% mentioned strong agreement from professionals and 46% some agreement with preventive measures [43].

The population’s attitude towards HPV vaccination, reflected by the perception of family doctors, was below expectations and more discouraging than that found in other studies, with only 1.0% of them mentioning a favorable attitude. The population’s attitude towards HPV vaccination is better in urban areas (*p* = 0.049), as reflected by the respondents from these areas, probably due to better educational levels and more frequent exposure to information.

An older study of Maier at al. (2015) mentioned that in the first vaccination campaign in 2008, 70% of parents were against vaccination [31]. Another two studies from 2022 reflected the attitude of the population in different way. Manolescu et al. reported that 10.51% of the general population were vaccinated against HPV, reflecting a higher degree of acceptance than that recorded in our study [44]. Voidăzan et al. found that 49.5% of adolescents and 52.3% of their mothers considered vaccination effective in preventing HPV infection [8]. A higher proportion of respondents from our study, 10.5%, held a rather favorable and 65.1% a partly favorable attitude, suggesting a higher proportion of positive attitude than that reflected by the 22% of doctors who specified that the population’s attitude towards HPV vaccination is partially or totally unfavorable.

A large proportion of family physicians from our study, 97,6%, practice vaccination according to the national calendar for children, and 90.0% of them usually vaccinate against HPV. A similar level of HPV vaccination in practices, 92.4%, was reported by Çevik et al. for family doctors from the WHO European Region [45]. We found a statistically significant difference (*p* = 0.004) between urban and rural areas regarding HPV vaccination, probably due to the increased involvement of family physicians in the adoption of this specific vaccination in city offices, the small number of respondents from rural areas, or the time constraints of rural doctors, who serve a larger number of patients.

We recorded a statistically significant difference (*p* < 0.001) according to area of residence regarding the number of HPV vaccine doses administered in 2022, with a median of 20 doses in urban areas and 9.5 doses in rural areas. This may indicate that there is less concern among rural physicians regarding HPV vaccination or a lower level of confidence. Data from the US suggest that fewer parents in rural areas report receiving a recommendation for HPV vaccine from a physician compared to those in urban areas [46].

We also found a statistically significant difference in recommending HPV vaccination over 18 years between urban and rural areas (*p* = 0.046), probably due to the small number of respondents from rural areas. Another study noted that this type of risk-based approach is generally used for vaccination recommendations, with the usual recommendation for HPV vaccination being reserved for younger ages, 11–17 years [47].

Doctors were the main healthcare professionals who provided educational information, at 97.6%, followed by nurses, at 45.9%. The most appropriate time for an educational discussion about HPV infection and HPV vaccination were noted to be during regular activity or when checking the vaccination schedules of children and adolescents newly enrolled in the office for 51.2% of the respondents. Educating patients at every opportunity when seeing a doctor seems to be the path to success for any intervention. Others chose to educate only upon the request of patients or their parents. This approach follows a well-known pattern of avoiding direct, potentially uncomfortable discussions and the fact that doctors underestimate the value that parents place on the HPV vaccine [48].

Another important aspect investigated in our study was related to the categories of patients who asked questions regarding HPV vaccination. Mothers, 85.6%, were the ones who asked the most questions. As mothers are the people who usually take care of children, it is expected that they have the involvement in the vaccination decision. Moreover, the female gender is the most affected by the consequences of HPV infection, like cervical cancer. Mothers’ interest in finding information about HPV and HPV vaccination has been much greater than that of other family members since the advent of the vaccine, as observed in studies that investigate the level of knowledge and attitude towards vaccination among the population [8,30,31,32,33,34].

Our findings from family doctors’ practices are slightly different from the data from a few years ago, when 46.2% of parents and 21.8% of mothers mentioned that they received information about HPV infection and vaccination and only 26.4% of parents, including 18.4% of mothers, requested information about this topic from their family doctor [8]. Mothers are now more involved in communication, an essential process that leads to increased trust in the doctor and, implicitly, to better acceptance of the HPV vaccine, as mentioned in a study conducted in Poland [49]. The provision of information by health professionals during consultations is a key factor contributing to the acceptance of HPV vaccination among parents and adolescents [50].

The main sources of information for family doctors were continuing education courses and events, followed by guidelines published by medical associations and medical society websites. The websites of international health organizations, such as the WHO, CDC, and ECDC, were used by 50.2% of respondents, with a statistically significant difference (*p* = 0.025) between urban and rural areas. Google was mentioned by only 10.0% of respondents, far fewer than we would have expected.

Our results are similar to those reported by Voidăzan et al. [8], who mentioned that 61.6% of doctors obtained information from specialized books, magazines, and brochures and 46.4% from the internet. Our results suggest a growing tendency for physicians to use verified educational materials from official, evidence-based sources to inform themselves.

The perception regarding the personal level of knowledge about HPV obtained expected results, with 60.3% of family doctors considering themselves to have good knowledge, 28.2% considering themselves to have very good knowledge, 11% partial knowledge, and only one doctor mentioning that they have poor knowledge.

Voidăzan et al. [8] found, in their study from 2022, that the level of information about HPV infection was satisfactory for 47.3% of doctors, good for 32.1%, and very good for 4.5%, while the level of information about HPV vaccination was satisfactory for 44.6% of physicians, good for 24.1%, and very good for 3.6%. Our study recorded an improvement in self-assessed knowledge levels that correlates with increased confidence and a higher frequency of vaccination recommendations, according to recent studies [51,52].

Although the level of knowledge seems to have improved, there were questions asked by patients that family doctors could not answer accurately. These were related to efficacy in boys, contraindications, safety, the evolution of the infection, and others. Statistically significant differences between urban and rural areas were recorded for contraindications (*p* < 0.001) and HPV distribution and transmission (*p* = 0.048). These aspects may be related to the high percentage, 58.4%, of family physicians who mentioned that they had not participated in training courses on HPV infection/HPV vaccination in the last 5 years.

Furthermore, only 24.4% of family physicians were exposed to information about HPV and HPV vaccination during their specialty training (residency, postgraduate studies). These percentages may be influenced by the time elapsed from the training program to the time of our study and the ability of each physician to accurately recall the information. However, the question of the need for a more extensive HPV training module during specialty training remains important.

Other studies have also highlighted the need to improve the training on HPV vaccination offered to resident physicians from different specialties, such as pediatrics, family medicine, obstetrics and gynecology, internal medicine, and dentistry, through the specialty training program, which should provide, in addition to theoretical knowledge, better communication and recommendation tools [11,41,47].

The essential information that needs to be included in appropriate training material for family physicians had different weights depending on gender and area of residence. Statistically significant differences were recorded in urban areas, with women being more interested than men in information on the distribution and transmission of HPV (*p* = 0.003), the evolution of HPV infection, and the burden of the disease (*p* = 0.005). This means that women doctors from urban areas need more scientific evidence about the disease than male doctors, probably due to aspects related to their own health and the desire to be able to help other women by providing the most appropriate advice.

In rural areas, women doctors were more interested than men in information on the benefits and risks of vaccination at 18 years of age and older (*p* = 0.033), advice for counseling parents and their children (*p* = 0.027), advice for counseling adolescents (*p* = 0.004), and advice for skeptical parents (*p* = 0.049). A possible explanation for this is that a discussion regarding a predominantly sexual pathology and its prevention is much easier to have between women, especially in the cultural context of rural areas. It is also possible that the rural population is more hesitant about HPV vaccination, as mentioned by Dumitra et al. [37], or even that family doctors are more hesitant or reluctant, as reported by Tron et al. [28], regardless of the administrative–territorial area in which they operate.

Other studies reported that female doctors had more favorable attitudes toward HPV vaccination and felt more comfortable discussing HPV-related topics with patients compared to men, due in part to their greater awareness of the role of HPV in cervical cancer and the influence of gender communication dynamics in healthcare [41,53].

Continuing medical education and undergraduate medical education were mentioned by Çevik et al. as solutions to ensure physicians recommended the HPV vaccine to their patients [45]. A 2025 systematic review mentions the need for personalized and desexualized communication training, based on a higher level of knowledge and communication skills, to manage vaccine hesitancy [51].

Healthcare professionals globally have reported various barriers to HPV vaccination, such as concerns about vaccine safety, the need for HPV vaccination, limited work time, and lack of parental support [44,51,54]. However, the strategy that is now most commonly used globally by healthcare professionals to recommend HPV vaccination is based on offering the vaccination option without insisting or trying to convince, inviting patients to discussions, using motivational interviewing, and involving them in decision-making [28,44,51].

Our results suggest that Romanian physicians face the same challenges and could benefit from training in effective communication strategies, especially in the current favorable context of expanded access to the HPV vaccine.

Our study was conducted specifically on a nationwide group of family physicians, who are the main vaccinators in Romania and frontline healthcare providers. It is the only study in recent years that has targeted this population and investigated such a wide range of aspects.

This study has the following potential limitations. A cross-sectional study design was used, and the sample was limited in its representativeness to respondents who have digital skills and were willing to complete the electronic questionnaire. The study included 209 family physicians nationwide, representing 2% of the total population of physicians in this specialty under contract with NHIH, and analyzed data collected in a limited time period of only 3 months. The number of rural respondents included in this study was small, which meant that some comparisons between residence areas, regarding certain components, were influenced by this factor. Additional and repeated studies are needed to consistently determine the level of knowledge, attitudes, practice, and training needs, as well as the challenges faced by family physicians, especially in the context of changes in the HPV vaccination strategy.

## 5. Conclusions

Romania has made significant progress in policies regarding access to HPV vaccination in recent years, but the coverage remains low. Family physicians from Romania consider themselves to have a strong level of knowledge about HPV vaccination, but less than half attended any training on HPV vaccination in the last 5 years, and one in five were not able to answer to some HPV-related questions coming from patients. Also, most consider other health professionals to have a moderate attitude towards HPV vaccination and the population is only “rather” or “partially” in favor of vaccination.

Therefore, awareness-raising actions for the general population—including parents and young people—are necessary in this regard, as well as continuous formal training for family doctors on HPV vaccination. These actions could lead to a steady increase in vaccination coverage among the priority population in the near future.

## Figures and Tables

**Table 1:** Demographic and administrative characteristics of family physicians.

Characteristic	Total (N = 209)	Urban (N = 138)	Rural (N = 71)	*p* Value
Gender *n* (%)				0.113
Females	179 (85.6%)	122 (88.4%)	57 (80.3%)	
Males	30 (14.4%)	16 (11.6%)	14 (19.7%)	
Age Me ^1^ (Q1–Q3)	56.0 (48.0–63.0)	55.0 (48.0–63.0)	57.0 (49.5–64.0)	0.321
Medical office type *n* (%)				0.128
Individual office	188 (90.0%)	121 (87.7%)	67 (94.4%)	
Grouped office	21 (10.0%)	17 (12.3)	4 (5.6%)	
Professional rank *n* (%)				<0.001
Consultant	144 (68.9)	109 (79.0%)	35 (49.3%)	
Specialist	54 (25.8%)	26 (18.8%)	28 (39.4%)	
General practice	11 (5.3%)	3 (2.2%)	8 (11.3%)	
FP which take care of children *n* (%)	203 (97.1%)	134 (97.1%)	69 (97.2%)	0.973
Number of children *n* (%) Me (Q1–Q3)	311.5 (137.0–529.0)	312.5 (107.5–589.5)	311.5 (185.0–487.5)	0.641
Access to NEVR *n* (%)	204 (97.6%)	134 (97.1%)	70 (98.6%)	0.504

^1^ Me = median; Q1 = 25th percentile; Q3 = 75th percentile; NERV = National Electronic Vaccination Registry; FP = family physicians.

**Table 2:** Attitude of family physicians regarding vaccination in general and HPV vaccination.

Attitude	Total (N = 209)	Urban (N = 138)	Rural (N = 71)	*p* Value
Respondents’ attitude towards vaccination in general				0.286
In favor	199 (95.2%)	131 (94.9%)	68 (95.8%)	
Rather in favor	9 (4.3%)	7 (5.1%)	2 (2.8%)	
Partly in favor	1 (0.5%)	0 (0.0%)	1 (1.4%)	
Respondents’ attitude towards HPV vaccination				0.158
In favor	195 (93.3%)	132 (95.7%)	63 (88.7%)	
Rather in favor	8 (3.8%)	4 (2.9%)	4 (5.6%)	
Partly in favor	4 (1.9%)	1 (0.7%)	3 (4.2%)	
Rather disapprove	1 (0.5%)	1 (0.7%)	0 (0.0%)	
Disapprove	1 (0.5%)	0 (0.0%)	1 (1.4%)	
Other doctors’ attitude towards HPV vaccination				0.749
In favor	54 (25.8%)	34 (24.6%)	20 (28.1%)	
Rather in favor	77 (36.8%)	53 (38.4%)	24 (33.8%)	
Partly in favor	69 (33.0%)	46 (33.3%)	23 (32.3%)	
Rather disapprove	2 (1.0%)	1 (0.7%)	1 (7.1%)	
Disapprove	1 (0.5%)	0 (0.0%)	1 (7.1%)	
Population’s attitude towards HPV vaccination				0.049
In favor	2 (1.0%)	2 (1.4%)	0 (0.0%)	
Rather in favor	22 (10.5%)	16 (11.6%)	6 (8.4%)	
Partly in favor	136 (65.1%)	96 (69.5%)	40 (56.3%)	
Rather disapprove	45 (21.5%)	22 (15.9%)	23 (32.4%)	
Disapprove	1 (0.5%)	1 (0.5%)	0 (0.0%)	

**Table 3:** Practical approach to vaccination and HPV vaccination.

Practice	Total (N = 209)	Urban (N = 138)	Rural (N = 71)	*p* Value
Vaccinate children according to the NIC	204 (97,6%)	133 (96,4)	71 (100.0%)	0.104
Usually provide HPV vaccination	188 (90.0%)	130 (94.2%)	58 (81.7%)	0.004
HPV vaccine doses inoculated in 2022; Me (Q1–Q3) ^1^	19.0 (6.0–40.0)	20.0 (10.0–47.8)	9.5 (3.0–24.0)	<0.001
Timing of educational discussion about HPV infection and HPV vaccination				
Periodical check-up	84 (40.2%)	58 (42.0%)	26 (36.6%)	0.450
I check the vaccination schedule for new children/adolescents and educate them or parents	107 (51.2%)	77 (55.8%)	30 (42.3%)	0.064
I educate children and adolescents of a certain age or their parents	96 (45.9%)	64 (46.4%)	32 (45.1%)	0.858
I routinely educate patients about HPV	107 (51.2%)	76 (55.1%)	31 (43.7%)	0.118
At the request of patients or their parents	84 (40.2%)	50 (36.2%)	34 (47.9%)	0.104
I don’t answer	1 (0.5%)	0 (0.0%)	1 (1.4%)	0.162
Vaccination of patients aged ≥18 years against HPV				0.046
Yes, but only upon request	37 (17.7%)	20 (14.5%)	17 (23.9%)	
Yes, for people at high risk of HPV-related cancer	4 (1.9%)	3 (2.2%)	1 (1.4%)	
Yes, I generally recommend	130 (62.2%)	96 (69.6%)	34 (47.9%)	
Yes, after conization of precancerous cervical lesions	3 (1.4%)	1 (0.7%)	2 (2.8%)	
Yes, for other reasons	6 (2.9%)	4 (2.8%)	2 (2.8%)	
No	28 (13.4%)	13 (9.4%)	15 (21.1%)	
I don’t answer	1 (0.5%)	1 (0.7%)	0 (0.0%)	
Providing information to parents, children and adolescents about HPV infection and HPV vaccination	207 (99.0%)	137 (99.3%)	70 (98.6%)	0.631
Who provides this information				
Doctors	204 (97.6%)	135 (97.8%)	69 (97.2%)	0.773
Nurses	96 (45.9%)	60 (43.5%)	36 (50.7%)	0.321
The interlocutor in the educational discussion about HPV vaccination				0.136
Mothers	100 (47.8%)	61 (44.2%)	39 (54.9%)	
Children/teenagers and parents together	74 (35.4%)	52 (37.7%)	22 (31.0%)	
Both parents	29 (13.9%)	22 (15.9%)	7 (9.9%)	
Adolescents	4 (1.9%)	1 (0.7%)	3 (4.2%)	
I don’t know/I don’t answer	2 (1.0%)	2 (1.4%)	0 (0.0)	
People who asked questions				
Mothers	179 (85.6%)	120 (87.0%)	59 (83.1%)	0.451
Fathers	0 (0.0%)	0 (0.0%)	0 (0.0%)	
Mothers and fathers equally	16 (7.7%)	10 (7.2%)	6 (8.5%)	0.756
Young adults	55 (26.3%)	41 (29.7%)	14 (19.7%)	0.120

^1^ Me = median; Q1 = 25th percentile; Q3 = 75th percentile; NIC = National Immunization Calendar.

**Table 4:** Source of information about HPV and HPV vaccination.

Source	Total (N = 209)	Urban (N = 138)	Rural (N = 71)	*p* Value
Internet				
Medical society websites	147 (70.3%)	92 (66.7%)	55 (77.5%)	0.106
Websites of international health institutions/organizations	105 (50.2%)	77 (55.8%)	28 (39.4%)	0.025
Online magazines	74 (35.4%)	50 (36.2%)	24 (33.8%)	0.728
Articles from scientific databases	114 (54.5%)	80 (58.0%)	34 (47.9%)	0.166
Vaccine manufacturers’ websites	76 (36.4%)	54 (39.1%)	22 (31.0%)	0.246
Google	21 (10.0%)	14 (10.1%)	7 (9.9%)	0.948
Print media				
Information notes/guides for doctors (e.g., published by medical associations)	147 (70.3%)	102 (73.9%)	45 (63.4%)	0.114
Vaccine manufacturers’ leaflets	136 (65.1%)	90 (65.2%)	46 (64.8%)	0.951
Scientific journals	56 (26.8%)	38 (27.5%)	18 (25.4%)	0.736
No	14 (6.7%)	9 (6.5%)	5 (7.0%)	0.887
Congresses or professional events				
Continuing education courses and events	170 (81.3%)	115 (83.3%)	55 (77.5%)	0.302
Informal collegial information exchange	80 (38.3%)	51 (37.0%)	29 (40.8%)	0.584
No	14 (6.7%)	9 (6.5%)	5 (7.0%)	0.887

**Table 5:** Topics that caused family physicians challenges in answering accurately.

Knowledge	Total (N = 209)	Urban (N = 138)	Rural (N = 71)	*p* Value
Efficacy in boys	59 (28.2%)	34 (24.6%)	25 (35.2%)	0.108
Contraindications	56 (26.8%)	49 (35.5%)	7 (9.9%)	<0.001
Safety/side effects	55 (26.3%)	33 (23.9%)	22 (31.0%)	0.271
Evolution of infection	43 (20.6%)	31 (22.5%)	12 (16.9%)	0.346
Benefits and risks ≥ 18 y	37 (17.7%)	27 (19.6%)	10 (14.1%)	0.326
Efficacy in girls	38 (18.2%)	25 (18.1%)	13 (18.3%)	0.973
HPV vaccines available	35 (16.7%)	24 (17.4%)	11 (15.5%)	0.728
Benefits and risks in boys aged 9 to 17	35 (16.7%)	24 (17.4%)	11 (15.5%)	0.728
Health consequences of HPV infections	35 (16.7%)	26 (18.8%)	9 (12.7%)	0.258
HPV distribution and transmission	32 (15.3%)	26 (18.8%)	6 (8.5%)	0.048
Benefits and risks in girls aged 9 to 17	25 (12.0%)	16 (11.6%)	9 (12.7%)	0.819
Diagnosis HPV infections	24 (11.5%)	18 (13.0%)	6 (8.5%)	0.324

**Table 6:** Importance of information about HPV and HPV vaccination (scored by type of information that needs to be covered by training).

Residential Environment		Urban		Rural	
Type of Information	Gender	M ± SD ^1^	*p* Value	M ± SD	*p* Value
HPV distribution and transmission;	Female Male	3.7 ± 0.5 3.3 ± 0.9	0.003	3.7 ± 0.4 3.6 ± 0.5	0.491
Evolution of HPV infection and burden of disease	Female Male	3.8 ± 0.4 3.4 ± 0.8	0.005	3.7 ± 0.5 3.6 ± 0.5	0.321
Diagnostic methods for HPV infection	Female Male	3.6 ± 0.5 3.6 ± 0.6	0.636	3.8 ± 0.4 3.6 ± 0.5	0.178
Health consequences of HPV infections	Female Male	3.8 ± 0.4 3.6 ± 0.6	0.108	3.8 ± 0.4 3.6 ± 0.5	0.140
Efficacy of HPV vaccination in girls	Female Male	3.8 ± 0.4 3.8 ± 0.6	0.621	3.8 ± 0.4 3.9 ± 0.4	0.901
Efficacy of HPV vaccination in boys	Female Male	3.8 ± 0.4 3.6 ± 0.7	0.135	3.7 ± 0.6 3.7 ± 0.6	0.942
Vaccination safety/side effects	Female Male	3.8 ± 0.4 3.7 ± 0.6	0.200	3.8 ± 0.5 3.9 ± 0.3	0.230
HPV vaccines available	Female Male	3.7 ± 0.5 3.7 ± 0.6	0.957	3.8 ± 0.4 3.6 ± 0.6	0.361
Contraindications	Female Male	3.8 ± 0.4 3.8 ± 0.6	0.867	3.7 ± 0.4 3.8 ± 0.4	0.711
Vaccination benefits and risks for girls 9 to 17 years	Female Male	3.7 ± 0.6 3.8 ± 0.6	0.695	3.8 ± 0.4 3.7 ± 0.5	0.453
Vaccination benefits and risks for boys 9 to 17 years	Female Male	3.7 ± 0.5 3.7 ± 0.6	0.994	3.8 ± 0.5 3.4 ± 1.1	0.072
Vaccination benefits and risks for ≥18 years	Female Male	3.7 ± 0.6 3.8 ± 0.6	0.689	3.8 ± 0.4 3.4 ± 1.1	0.033
Advice for counseling parents and their children	Female Male	3.7 ± 0.5 3.9 ± 0.5	0.268	3.8 ± 0.4 3.5 ± 0.7	0.027
Advice for counseling adolescents	Female Male	3.8 ± 0.4 3.8 ± 0.6	0.812	3.8 ± 0.5 3.3 ± 0.9	0.004
Counseling sessions with skeptical parents	Female Male	3.7 ± 0.6 3.5 ± 0.9	0.212	3.7 ± 0.6 3.3 ± 0.8	0.049
Counseling sessions with skeptical adolescents	Female Male	3.7 ± 0.6 3.4 ± 0.9	0.122	3.7 ± 0.6 3.3 ± 0.8	0.077
Conversation-guiding techniques	Female Male	3.6 ± 0.6 3.5 ± 0.8	0.603	3.5 ± 0.8 3.3 ± 1.0	0.417
Dealing with difficult counseling situations	Female Male	3.6 ± 0.6 3.6 ± 0.6	0.971	3.5 ± 0.8 3.1 ± 1.1	0.087
Case studies/clarification questions from practice	Female Male	3.6 ± 0.7 3.5 ± 0.7	0.750	3.5 ± 0.7 3.2 ± 1.0	0.164
Culturally sensitive counseling about HPV vaccination	Female Male	3.5 ± 0.7 3.4 ± 0.9	0.414	3.6 ± 0.7 3.4 ± 1.0	0.350
Procedures applied for positive PAP tests/HPV testing	Female Male	3.6 ± 0.6 3.4 ± 0.9	0.297	3.6 ± 0.7 3.5 ± 0.9	0.643
Treatment of HPV-associated carcinomas/precursor lesions	Female Male	3.6 ± 0.6 3.3 ± 0.8	0.101	3.6 ± 0.6 3.4 ± 1.1	0.242
Treatment of anogenital warts	Female Male	3.6 ± 0.6 3.4 ± 0.7	0.180	3.6 ± 0.6 3.4 ± 0.9	0.295

^1^ M = mean, SD = standard deviation; scores as follows: very important = 4; important = 3; neutral = 2; less important = 1; not at all important = 0.

## Data Availability

The original contributions presented in the study are included in the article, further inquiries can be directed to the corresponding author.

## Abbreviations

The following abbreviations are used in this manuscript:

CC	Cervical Cancer
FP	Family Physicians
HPV	Human Papilloma Virus
NHIH	National Health Insurance House
NIC	National Immunization Calendar
